# The Role of Molecular Biology in the Biomonitoring of Human Exposure to Chemicals

**DOI:** 10.3390/ijms11114511

**Published:** 2010-11-12

**Authors:** Balam Muñoz, Arnulfo Albores

**Affiliations:** Departamento de Toxicología, Centro de Investigación y de Estudios Avanzados del IPN, Av. IPN 2508, México, DF 07360, Mexico; E-Mail: rbmunoz@cinvestav.mx

**Keywords:** molecular biology, occupational toxicology, biological monitoring

## Abstract

Exposure to different substances in an occupational environment is of utmost concern to global agencies such as the World Health Organization and the International Labour Organization. Interest in improving work health conditions, particularly of those employees exposed to noxious chemicals, has increased considerably and has stimulated the search for new, more specific and selective tests. Recently, the field of molecular biology has been indicated as an alternative technique for monitoring personnel while evaluating work-related pathologies. Originally, occupational exposure to environmental toxicants was assessed using biochemical techniques to determine the presence of higher concentrations of toxic compounds in blood, urine, or other fluids or tissues; results were used to evaluate potential health risk. However, this approach only estimates the presence of a noxious chemical and its effects, but does not prevent or diminish the risk. Molecular biology methods have become very useful in occupational medicine to provide more accurate and opportune diagnostics. In this review, we discuss the role of the following common techniques: (1) Use of cell cultures; (2) evaluation of gene expression; (3) the “omic” sciences (genomics, transcriptomics, proteomics and metabolomics) and (4) bioinformatics. We suggest that molecular biology has many applications in occupational health where the data can be applied to general environmental conditions.

## 1. Introduction

**Figure 1.**
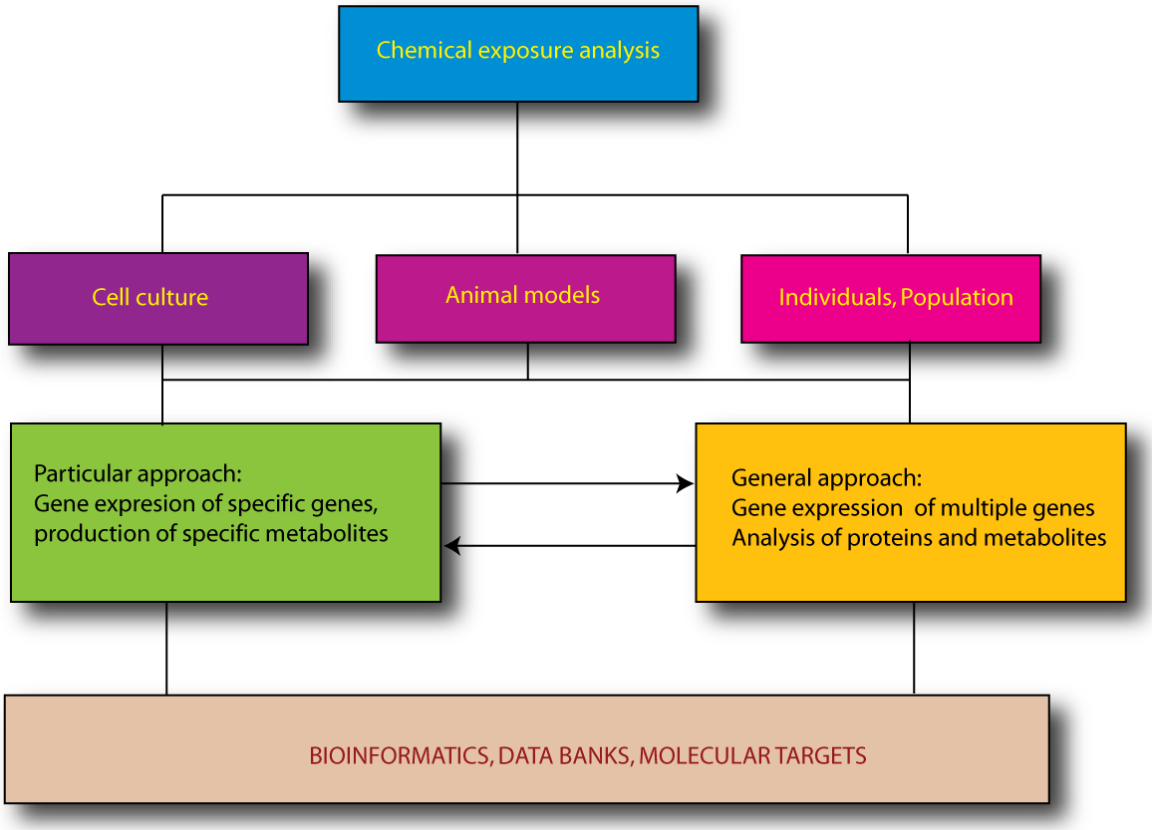
The role of molecular biology in chemical exposure analysis. A representative scheme shows two *in vitro* study approaches for specific genes or for gene groups.

In everyday and occupational environments there are ubiquitous noxious chemicals that can be hazardous to human and wildlife health. Recently, molecular biology techniques have received greater attention for their potential to contribute to determining the presence of toxic substances in the environment that may be detrimental to health. Accurate human exposure studies require sensitive methods capable of evaluating dose-response relationships, particularly in cases of low level exposure to noxious agents. Here we describe some of the most common tools used in molecular biology that can be applied to evaluate the effects of chemical exposure in occupational settings. These tools are: (1) use of cell culture; (2) evaluation of gene expression; (3) “omic” techniques and (4) bioinformatics (Figure 1). Furthermore, we discuss applications of molecular biology techniques for biomonitoring the effects of chemical exposure on human health. Recently, the use of new molecular biomarkers has changed the “classical” biochemical or chemical approach of evaluating damage after chemical exposure. Biomarkers or biological indicators are measurable changes at the physiological, biochemical or morphological level in the affected organisms after toxic exposure. These biomarkers are used to detect exposure, determine the biological consequences of the exposure, and to evaluate initial or intermediate stages of pathological processes, identifying individuals that may be sensitive to specific chemicals. These molecules are then classified as exposure, effect or susceptibility biomarkers, respectively. Therefore, the purpose of developing new biomarkers is to estimate the relationship between environmental or occupational chemical exposures and their subsequent effects in individuals or open populations.

## 2. Cell Cultures

In molecular biology, cell culture techniques are an invaluable tool for analyzing physiological and molecular processes. Cell culture is useful for evaluating chemical damage and to establish cytotoxicity. Cell culture offers several advantages over animal studies. For instance, cell cultures are easy to handle and to keep under a controlled environment in an incubator. In addition, cell cultures allow for testing different compounds simultaneously and screening for chemical toxicity can be performed at a lower cost than in animal models.

The use of cell culture systems in toxicology has gained wide acceptance in cell-specific and metabolic studies. There are several models frequently used in toxicology including: primary cell cultures, immortalized cell lines, stem cells, and 3D cultures [1].

### 2.1. Primary Cell Culture

Several recent studies have used cells derived from tissues and organs to test their response to chemicals. The enormous advantage of primary cell culture is the direct evaluation of cytotoxicity and related mechanisms in normal cells. Hepatocytes are the most commonly used primary culture in toxicology. For example, human hepatocytes have been used to show the heterogeneity of CYP expression in the human liver [2]. However, the *in vitro* phenotypic instability of these cells, the irregular availability of fresh human liver and the high batch-to-batch variability of hepatocyte preparations obtained from donors complicate their use in routine testing [3]. In a recent study lung derived cells were used to evaluate the expression of CYP isoforms following exposure to PAHs or nitrosamines. Expression levels of these enzymes were then used as effect biomarkers [4]. In another study the relevance for occupational health risk assessment of engineered nanoparticles in lung toxicity is addressed. In this study, primary cells, such as epithelial and alveolar macrophages, are used to evaluate cytotoxicity by measuring cytokine levels [5]. Primary cells such as lymphocytes are useful to evaluate toxicity of carcinogenic compounds like benzene [6].

### 2.2. Cell Lines

Various cell models have been fully characterized and collections can be obtained from research organizations like the American Tissue and Cell Collection (ATCC). Here representative cell lines for each organ or tissue can be collected for specific purposes, making it possible to evaluate the metabolic or genetic effects of a chemical on specific types of cells. Another advantage of these cultures is that they can be engineered at specific genes sites that affect certain molecular processes. For instance, hepatoma derived cells engineered to contain the luciferase gene respond to dioxin stimulus. These cells are called CALUX and are also useful for evaluating environmental impact [7]. Another example is the use of immortalized cell lines like A549 (a lung cell line) to evaluate toxicity to aromatic compounds [8]. An additional study suggests that immortalized keratinocytes lines (RHEK-1, HaCaT, and NM1) can be used to evaluate toxicity of chemicals such as lead, cadmium or arsenic [9]. Moreover, the role of transcriptional regulation and cell signaling in drug-exposed cells offers new study opportunities [10]. As mentioned before, engineered cell cultures are useful to test the role of exposure to a chemical agent in the over-expression or down-regulation of certain proteins. Cell cultures could be engineered by plasmid or siRNA transfection, virus transduction or biobalistics. Genetic engineering of cells is important for analyzing the genotoxicity associated with specific xenobiotic-metabolizing enzymes and it is useful for elucidating metabolic activation and inactivation processes. In a recent work, a cell line derived from Chinese hamster V79 was modified to express both human cytochrome P450 (CYP) 2E1 and human sulfotransferase (SULT). However, although these cells activate various important pro-genotoxicants, they have been observed to elevate spontaneous gene mutation frequencies [11]. The use of the V79 cell line in the study of cytochrome P450 using siRNA technology is another example of the importance of cell lines in toxicology [12].

### 2.3. Stem Cells

The ability to proliferate without limits and easily differentiate into different cell types are advantages of stem cell cultures over other systems (*i.e.*, primary cells). As a result of these advantages stem cell cultures have generated great interest as potential tools for toxicological screening. Stem cell culture systems help to evaluate differentiation or transdifferentation of stem cells to non-related tissues in the presence of teratogenic chemicals [1]. In other cases, these cells are induced to differentiate into hepatocytes or cardiomyocytes to test chemical agents [13]. However, the use of stem cells from embryonic tissue, human embryonic stem cells (hESC), remains controversial. Recently new perspectives have been opened by the use of induced pluripotent stem cells (iPSC), generated by the virus-mediated expression of only four genes and shown to demonstrate characteristics similar to those of hESC [14]. Phthalates are synthetic ubiquitous compounds distributed into the environment. Humans are continually exposed to phthalates due to their use in plastics and other common consumer products. It is worth mentioning that, due to their hormone profile, women are particularly susceptible and have a unique exposure profile to phthalates, raising concern for potential health hazards. In a recent study, stem cells were used to analyze cytotoxicity and gene expression in phthalate treated cells. Results from this study are useful to understand potential mechanisms of toxicity in human embryos [15]. In another study, researchers used mouse embryonic stem cells to evaluate both the neuropathy target esterase as a biomarker of embryotoxicity processes and its role in differentiation [16].

### 2.4. Three Dimensional-Cultures

In three-dimensional (3D) cultures, cells are seeded in a protein matrix. This matrix allows a well defined geometry and establishes a similar environment to that of a tissue (an *in vivo-*like niche), while the 3D culture promotes cellular interaction between cells and the matrix. Several polymers useful for matrix constitution include: agarose, collagen, gelatin and matrigel [17]. Another advantage of this model is the possibility of establishing a co-culture or promoting differentiation. In fact, a microarray using 3D culture has been successfully employed to evaluate toxicity of chemical compounds by analyzing cytochrome P450 induction [18]. Modeling 3D cultures is a useful tool to improve the predictive value of cell-based assays for safety and risk assessment studies [19]. In a recent study, HepG2 cells cultured in a 3D matrix were observed to exhibit an increase in hepatocyte-specific functions, including drug-metabolizing enzyme activities, making this model suitable for evaluating toxicity *in vitro* [20]. Furthermore, 3D cultures have been established to test cellular and molecular effects on occupationally-exposed individuals [21]. Finally, it has recently been suggested that 3D cultures can reduce the cost of evaluating the toxic potential of new drugs or xenobiotics [22], and the toxicity of nanoparticles [23].

## 3. Evaluation of Gene Expression

By using molecular biology, we can evaluate gene expression by measuring mRNA or protein levels. Several routine techniques, such as polymerase chain reaction (PCR), Enzyme-Linked ImmunoSorbent Assay (ELISA) or microarrays have an extensive application in chemical exposure monitoring. Furthermore, some of these techniques can be performed at a lower cost and in less time in comparison to other classical techniques. In fact, the use of real time PCR has had an enormous impact in toxicology due its accuracy and versatility. Recently, occupational toluene [24] exposure was evaluated using CYP2E1 expression as a biomarker by real time PCR. ELISA has become another useful tool for rapidly detecting and quantifying proteins in multiple samples. Finally, microarrays can detect expression of several genes simultaneously and in specific conditions. Recently, microarray technology has been used to evaluate gene expression of four toxic components: oxythioquinox (a quinoxaline pesticide); malathion (an organophosphate pesticide); di-n-butyl phthalate (a chemical commonly found in personal care and cosmetic items); and benzo[*a*]pyrene (BaP, an environmental and occupational carcinogen). The study found that exposure to any of these chemicals produced an increased expression of metabolic enzymes or genes related with fertility. In particular, BaP altered expression of two cytochrome P450 isoforms [25].

### 3.1. qRT PCR

Quantitative analysis of changes in molecular targets plays an important role in addressing scientific questions in molecular toxicology and epidemiology, and results are used in human risk assessment. One of the emerging technologies used to analyze these molecular targets is quantitative real-time polymerase chain reaction (qRT PCR). Three applications of qRT PCR for genetic and molecular toxicology are: quantification of gene expression, detection of genetic polymorphisms, and quantification of chromosomal DNA deletions [26,27]. Gene expression analysis has become an approach to identify biomarkers and understand mechanisms of toxicity. For example, Cyclin D1 expression is an effective biomarker for evaluating cell proliferation in response to a particular toxic agent. In another study qRT PCR is used to validate microarray studies. In this work, gene expression profiles of rat cerebellum were evaluated in response to mixtures of methylmercury, polychlorinated biphenyls and organochlorine pesticides. This technique revealed differential expression in genes that regulate key processes in neuronal activity and the authors suggest that these genes are also affected in exposed individuals. However, genotyping methods are required since some experiments have revealed various genetic polymorphisms that modify effects of chemical exposure in humans. One of these methods is TaqMAMA, an RT PCR-based genotyping technique that combines the strengths of TaqMan with the allelic discrimination of the mismatch amplification mutation assay (MAMA). A recent report uses qRT PCR to measure gene expression of human kidney cells exposed to uranium. In this paper, the level of the SPP1 gene is greatly increased and the author suggests its use as a biomarker to evaluate occupational exposure [28,29].

### 3.2. ELISA

The ELISA technique is appropriate for the general assessment of immune, metabolic and genotoxic responses resulting from chemical exposure. This technique is highly sensitive and can be quantitative. Nowadays, because of the wide variety of commercial kits, the assay can be easily incorporated into industrial drug and chemical efficacy testing programs. Some of these kits are useful to measure metabolites as a result of chemical exposure. For instance, exposure to tobacco smoke can be detected by measuring nicotine and its metabolite cotinine. Due to the long half-life, cotinine has been considered to be a reliable biomarker for smoking status and several suppliers have ELISA kits that detect and measure cotinine levels. In other cases, ELISA kits are used to measure protein levels serving as biomarkers. Upon investigation, some proteins involved in the inflammatory response, specifically lipoxygenase and leukotriene, increase their expression with exposure to arsenic [30]. Another common use of ELISA in evaluation of chemical exposure effects is to determine induced cellular damage. By using ELISA, the levels of proteins involved in apoptosis or cell viability can be measured [31]. A recent study suggests that ELISA may be a valuable tool for evaluating cytokine levels in workers exposed to organic dust [32]. In another study ELISA was used to measure diverse cytokine levels in response to isocyanates (a group of low molecular weight aromatic and aliphatic compounds with diverse industrial applications) in isolated lymphocytes from volunteers [33]. In both studies cytokine levels were found to increase, suggesting that chemical exposure has important immunotoxic consequences. Finally, the ELISA technique has been applied to detect adducts between chemicals and biologic molecules such as DNA or proteins [34,35].

### 3.3. Microarrays

Recently, the use of microarrays has found several applications in chemical exposure evaluations, making the application of gene expression analysis in toxicology a mature science. Although this technology remains expensive, the field has rapidly progressed and gene expression profiling is now being used in screening for toxicity of new and existing chemical compounds and their effects on organisms [36]. Sen, Mahadevan and DeMarini (2007) did an excellent review of the analysis of gene expression in the presence of complex mixtures (cigarette smoke, urban air or diesel exhaust particles) and common contaminants. They found that complex mixtures generally induced expression of genes involved in oxidative stress response and xenobiotic metabolism [37]. They observed the expression of two genes, heme oxygenase 1 and CYP1A1, to be induced by each of the complex mixtures texted. For instance, in a recent study microarray technology was used to analyze gene expression of alveolar cells exposed to diesel particle extract. This work revealed multiple up-regulated genes involved in metabolism, cell cycle, apoptosis and antioxidant activities, among others [38]. In another study, the use of microarray analysis from the global gene expression profile of rat blood identified distinct gene expression markers capable of detecting and distinguishing if hepatotoxicity and neurotoxicity could be induced by different chemical agents [39]. Another common occupational health hazard is exposure to radiation. Microarray technology has been used to investigate its effect by analyzing gene expression profiles of lymphocytes isolated from radiation exposed-workers. Gene expression analysis revealed statistically significant transcriptional changes in a total of 78 genes (21 up-regulated and 57 down-regulated) involved in several biological processes including DNA repair, cell proliferation and stress response [40]. Finally, microarray technology was used to observe that exposure to polycyclic aromatic hydrocarbons in HepG2 and A549 cells altered several important pathways and genes including xenobiotic metabolism, oxidative stress response, proliferation, protein degradation and ion transportation, among others [41].

## 4. “Omics” in Analysis of Chemical Exposure

This new molecular approach to studying gene-environment interactions is referred to as toxicogenomics. It combines genomics, transcriptomics, proteomics, metabolomics and bioinformatics with conventional toxicology and pathology to analyze effects of occupational exposure on thousands of genes and proteins simultaneously. The creation of databanks and the development of new methods to analyze multiple genes or products simultaneously represent a multitude of new opportunities in toxicological studies and occupational health.

### 4.1. Genomics and Transcriptomics

At the forefront of these emerging technologies is the use of functional genomics or analysis of molecular perturbations to be assayed by microarray technology. Genomic biomarkers of toxicity have recently been identified for a wide variety of toxicants including nephrotoxic agents, testicular toxicants, and for keratinocyte proliferation in a papilloma murine skin model. Microarrays have also enabled high-throughput screening (HTS; a method that allows the rapid performance of millions of biochemical, genetic or pharmacological tests) of chemicals for potential toxicity. Increased sensitivity to detect molecular signatures or “fingerprints” is associated with a particular class of chemicals or toxic responses in both *in vitro* and *in vivo* systems [42]. Benzene, a potentially noxious substance, is a common solvent used in the manufacturing industry. The availability of biomarkers to detect benzene exposure has increased by using gene profiling “omics” techniques. The analysis of peripheral blood cells from benzene exposed workers revealed that more than 100 genes have differentially altered expressions [43].

### 4.2. Proteomics

The applications of proteomics in toxicology and analysis of chemical exposure can be divided into two classes: investigative studies, and screening or predictive toxicology. Investigative studies may help to identify new molecular targets for toxins and provide novel clues into mechanisms of action. Predictive toxicology refers to the screening of novel compounds and the study of structure-activity relationships (SARs) within a group. The use of proteomics in screening and predictive toxicology has two principal applications: establishing relationships between toxic effects and defining protein molecular biomarkers, *i.e.*, identifying toxicological biomarkers, and recognition of specific response patterns. Completely mapped proteomes of important biological fluids, such as blood serum or urine, will be crucial for biomarker development. Furthermore, identification of specific xenobiotic-protein adducts by proteomics will provide insights in immune, cellular and molecular responses to toxicants. The focus of proteomics ranges from global protein analysis to a more specific level of protein analysis. Some of the more common techniques used in proteomics include 2D gel analysis, mass spectrometry and chromatography. For instance, a recent study evaluates the proteomic profile of urine from uranium-exposed rats. Uranium is used in several industries and is a well known nephrotoxin. In normal urine there are, at least, 102 proteins; expression of fourteen of these proteins was significantly different in treated rats [44]. Another example is the proteomic analysis of ovaries from rats exposed to tetrachlorodibenzo-p-dioxin (TCDD). TCDD is a dioxin-derivative found at low concentrations in industrial wastewater. This compound induces an endocrine disruption that affects the reproductive system. In this study, the matrix-assisted laser desorption/ionization (MALDI) tandem mass spectrometry showed distinct changes in the levels of several proteins that are relevant markers for TCDD toxicity [45]. Proteomics is also useful to evaluate protein profiles from cell cultures. In a recent paper, epithelial cells were treated with *trans*,*trans*-2,4-decadienal (tt-DDE), a specific type of dienaldehyde that is abundant in heated oils or cooking oil fumes. Proteomic analysis by 2D electrophoresis and mass spectrometry revealed that two proteins DJ-1 (an oncogenic protein responsive to oxidative stress) and cofilin (a member of the actin depolymerization factor (ADF)/cofilin family and essential protein for cell viability) are suitable biomarkers for tt-DDE exposure [46].

### 4.3. Metabolomics

Metabolite prediction is based on a reaction-mechanism-based methodology, which applies fundamental organic and enzyme chemistry. This tool can be used to complement experimental studies of chemical mixtures, aid in risk assessment and help to understand the effects of complex chemical mixtures. Chemical agents at low dose levels can produce biological responses in protein expression patterns (proteomic responses) or alterations in sensitive metabolic pathways (metabolomic responses). Metabolomics is advantageous because (a) it analyses the last step in a series of changes following a toxic insult; (b) many metabolites have a known function and (c) changes are detectable in blood. Recently, a study revealed that metabolic profiles from butylbenzyl phthalate (BBP) treated-rats exhibited different patterns in comparison to non-treated individuals. Worker exposure to BBP was estimated at 143 and 286 μg kg^−1^ body weight per day by recovering metabolites from urine and considered for differences in dose, vehicle and sex. The authors suggested that the application of metabolomics was useful to understand the mechanistic link between low levels of environmental or occupational exposure and disease/dysfunction in a human population. Moreover, the use of biological fluids such as urine simplifies sample processing and analysis [47]. The complete integration of several study branches is called systems toxicology [48] of which the final aim is to improve human health using and complementing data obtained from individual disciplines.

## 5. Bioinformatics

**Figure 2.**
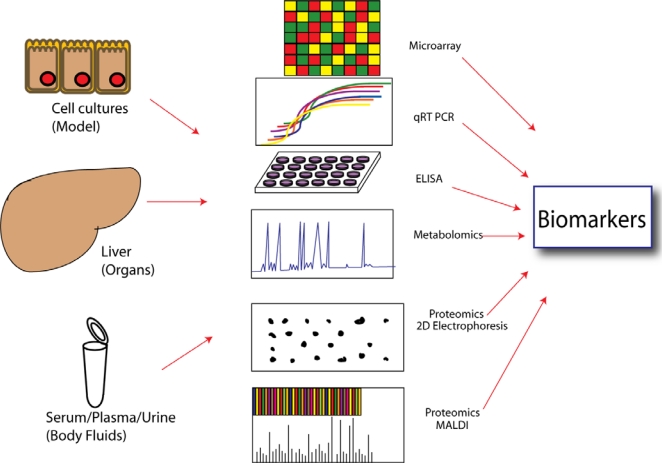
Development of new biomarkers to evaluate chemical effects in biological systems. An integrative approach using molecular biology tools include results from microarrays, proteomic analysis, metabolomics, and gene expression that will be useful to create databanks and develop new biomarkers of exposure. Adapted from Wetmore (2004) [50].

Computational or *in silico*, analyses lend substantial predictive power to evaluate occupational risk in addition to traditional methods of experimental biology. In this way, bioinformatics has become a powerful tool in toxicological research. Moreover, bioinformatics allows creation and administration of large gene or protein databanks. Methods of data integration for orthogonal “omics” datasets fall broadly into two categories: (a) biology-driven strategies and (b) data-driven strategies. Biological interpretations tend to be more hypothesis-driven and take advantage of a contextual understanding of what is currently known about the toxicity mechanism [42]. Some toxicants can be preferentially targeted to specific organs, tissues, or cell types due to their affinity for target proteins, often expressed in a tissue-specific manner or expressed at higher levels in the target tissue. The expression of these proteins and their mRNAs has traditionally been examined experimentally using immunological, hybridization, or PCR-based detection methods. However, with the advent of expressed sequence tags (ESTs) it is now possible to qualitatively and quantitatively measure mRNAs of target genes in a tissue and stage-specific manner by searching and counting ESTs in public databases. The development of software to analyze gene clusters is another powerful technique. SAMURAI is a program which organizes gene clusters from a library and complements the information with a chemical toxicity dataset, allowing for the assessment of the up/down-regulation of gene sets [49]. Another example of integrative bioinformatics is the Chemical Effects in Biological Systems (CEBS) [50], the integration of a DNA microarray, including proteomic and metabolomic studies, with toxicology data and the queries across “omic” platforms that generate new potential biomarkers of exposure (Figure 2). A recent study reports the development of a program called ebTrack. This bioinformatic tool integrates several databases and predictive tools that are critical for environmental and occupational health research. These health risk analysis tools include the Modeling ENvironment for TOtal Risk studies (MENTOR) for source-to-dose exposure modeling and the DOse Response Information ANalysis system (DORIAN) for health outcome modeling [51]. Another popular database is the comparative toxicogenomics database (CTD) which helps to understand the chemical-gene-disease relationship [52,53]. Profiles of chemical effects on cells (pCEC) is a recent database which offers a new tool to evaluate risk [54]. This tool is able to manage data from DNA microarrays, proteomics or metabolomics and integrate this data with models based for the “coupled” toxicokinetics and toxicodynamics of contaminants of concern and their mixtures. These tools make the search for potential biomarkers in occupational health easier and faster.

## 6. Molecular Biology in Evaluation of Risk to Chemical Exposure

Risk assessment focuses on maintaining exposure to environmental agents below the level at which significant perturbations of these pathways occur. The most important exposure assessment is the biologically effective dose, which is, the dose available at the site of the target tissue in the body. The biologically effective dose can typically be measured as the intact chemical or a biomolecular adduct in the target tissue. Blood and urine are, by far the most common matrices. Human Biomonitoring (HBM) can be done for most chemical substances and is currently a focus of the worldwide discussion of environmental medicine. This procedure has a particularly effective for monitoring metals, PAHs, phthalates, dioxins, pesticides, as well as for aromatic amines, perfluorinated chemicals, environmental tobacco smoke and volatile organic compounds. Determination of protein adducts (especially Hb-adducts) or DNA adducts are still better means to estimate cancer risk than measuring genotoxic substances and their metabolites in the human body fluids [55]. HBM measurements have been long used for evaluating occupational exposure to a variety of industrial chemicals. Molecular biology techniques are therefore a valuable tool for measuring chemical exposure.

### 6.1. Polycyclic Aromatic Compounds

Human exposure to PAHs can occur via inhalation, skin deposition/contamination or orally. Biomonitoring in the work environment is usually conducted using urine and blood samples. PAHs can be oxidized to electrophilic compounds that can react with macromolecules such as DNA or proteins to give adducts. Detection of these adducts is an important tool to evaluate risk as well as the level and degree of chemical exposure to PAHs. DNA adducts are considered biomarkers of effect in a specific target organ. Other common biomarkers to PAHs exposure are chromosomal aberrations (CA), micronuclei (MN) and mutations which are indicative of irreversible effects. The main human exposure groups to PAHs are workers from the coal, oil, asphalt or aluminum industries along with individuals exposed to everyday environments [56].

### 6.2. Metals and Non-Metals

These materials represent an important source of dangerous exposure in humans, particularly in chemical industry workers. Metals, such as iron, lead, cadmium, chromium or non-metals such as arsenic and asbestos are some of the most commonly used raw materials in industry. Although the immediate and long-term effects of continuous exposure to these materials are widely known a recent review describes new developments in cadmium (Cd) exposure analysis [57]. Some biomarkers to detect Cd include albumin, beta-2 microglobulin, retinol binding protein and glucosamine-6-phosphate deaminase (NAG/NAGB). However, Cd causes damage in several organs and tissues. For this reason new biomarkers and molecular techniques to detect metals are needed. It is commonly known that exposure to metals reduce lymphocyte levels. Metals and metal compounds responsible for reduced circulating antibody titers are lead, cadmium, methylmercury, arsenic, nickel, chromium and platinum [58]. Analysis of antibody titer is a suitable assay to measure chemical exposure.

### 6.3. Radiactive Materials

Nuclear industry workers and health personnel that use radioactive material in addition to people exposed accidentally to radiation need biomonitoring to measure their level of exposure. Chromosome aberration analysis is the conventional mean for assessing radiation exposure. A recent report suggests the real-time fluorogenic 5’-nuclease, or TaqMan polymerase chain reaction assay is being used to identify radiation-responsive molecular biomarkers, including gene expression targets and DNA mutations [59].

## 7. Conclusions

Exposure to harmful substances can be expected in several areas of work and there is a causal connection between work-related exposure and disease. There are two measurement strategies in environmental and occupational medicine to record the health risk to the general population and to exposed employees: environmental monitoring and biomonitoring. Although environmental monitoring has been used extensively, biomonitoring is a relatively new way of recording and quantifying environmental and occupational loads. The selection of the biomonitoring parameter for each case must be based on the specific toxic effect of the foreign substance. Biomonitoring studies cover workers from countries worldwide and from various industries including: oil, coal mines, agriculture, *etc.*, and for different markers and noxious compounds. Literature on the biomonitoring of specific populations is growing and through molecular biology tools this process is now much faster and more accurate. Molecular biology techniques have become useful and powerful tools in occupational disease and chemical exposures studies. The greatest contributions thus far have involved understanding mechanisms and modes of action.
